# Sex and Contagion

**Published:** 1874-11

**Authors:** James Nevins Hyde

**Affiliations:** Chicago


A MONTHLY RECORD OF

*Medicine, Surgery and the Collateral Sciences.*

EDITED BY J. ADAMS ALLEN, M.D., LL.D.; AND WALTER HAY, M.D.

VOL. XXXI. — NOVEMBER, 1874. — NO. 11.

### Original Communications.

ARTICLE I.—*Sex and Contagion.* By JAMES NEVINS HYDE, M.D., Chicago.

Pathological life, according to a recent writer,* is "consequent upon increased, diminished, or otherwise altered velocity† of molecular material, changing the arrangement of such material, interfering with the function which depends upon such arrangement, and, generally, inducing a greater or less modification of external appearance."

Without pausing to accept or reject the theory which such a definition involves, it may be applied in such a manner as to do no violence to generally received opinions.

There is an average condition of health, to the one side and the other of which, the pendulum of life swings from minute to min-

* McElroy. Cincinnati Lancet and Observer, August, 1874.

† The word "velocity" is, of course, intended to express, in its mechanical sense, the relation of motion to time. The rate of motion may be inappreciable, or appreciable only differential observations.

ute and from hour to hour, within certain prescribed physiological limits. In surpassing these limits, either to the right or left, the pendulum reaches an intermediate space, where the regions of health and disease are illy defined. A still wider swing encroaches upon the borders of that region which is allotted to pathology.

It will suffice for present purposes, if it be conceded that the greater deviation from this physiological average, is that which most nearly coincides with the pathological extreme. It should then follow that the study of the widest of these deviations must throw a flood of light upon pathogenesis.

The slightest of these variations are the transient phenomena of animal life, well worth the attention of the philosopher and the physician—the widest, offer them the surest basis upon which to found an explanation of malignant disease.

What are the widest of these deviations?

In the vegetable world, it is clear that plants sprout, flourish and die, for one special purpose — the production of a seed through which they may restore their kind. In a materialistic point of view merely, animals (including man) live to the same end—the perpetuation of their species. From infancy to puberty, the human male animal is sustained in order that he may finally secrete an organic cell, which shall possess the marvellous power of fecundating another and similar cell. This latter is provided by the female, who has survived for that main purpose nearly a quarter of a century. At the time of fruitful congress these cells are liberated,* endowed with a certain mode of motion, and there results a new being. Many individuals of a lower species, perish immediately after this supremest act of their short lives,† the liberated cells being extruded from their bodies, and placed in such media, or wrapped in such envelopes, that the new beings are therein fed and protected until sufficiently mature to feed and protect themselves. The lives of the human parents are, however, prolonged beyond this period, not only in order that they may be enabled to feed and protect the most helpless of offspring, but

* Consult Flint's Physiology of Man, Vol. 5, pp. 293–6, for evidence as to the probability that, apart from the menstrual molimen, the graafian follicle is ruptured during, or in consequence of, sexual intercourse.

† *E. G.*, the bombycidæ.

also that repeated acts of procreation may evoke other new organisms to life.

Now, a general survey of this cycle of events, might justify the conclusion that the maximum of that sum of velocities which we call life, would occur at the precise moment when the new organism was to be impressed with momentum. This, therefore, should be the date when those changed velocities originate, which have been supposed to be pathological.

However it may be with the parents, it is true that at this time the new life is endowed with all its hereditary tendencies to disease and death.

So far as regards the parents, however, the process of generation is not yet perfected. The female is obliged to furnish food and protection, first for the ovum and then for the fœtus, in a receptacle provided for that purpose, during a comparatively fixed interval, till the new being is capable of sustaining a separate existence. By means of a placenta and umbilical cord, the fœtus is attached to the mother, somewhat as a cherry is fastened to a fruit-bearing limb, and the maternal and fœtal bioplasts are thus commingled in continuous vascular channels.

The physiological velocities of the mother are thus again communicated to the child.

But there is a limit to this phase of life. At a determined instant, muscular energy intervenes, and plucks the mature fruit from the womb, in such a manner as if the stem of the cherry were torn from the limb, and the cherry itself torn from its stem. Here a physiological amputation is effected, and two physiological wounds are inflicted—one upon the matrix of the mother, and the other upon the umbilicus of the child.*

The maximum of their common velocity has been split. There are two resultants, in each of which must necessarily occur a change in the mode of motion.

Thereafter, if the natural process be completed, the child is

* These words are intended merely to call attention to the *physiological* phase of parturition, and not to imply assent to the doctrines of the localists, who look for the origin of puerperal diseases, to these lesions. Trousseau and Simpson, have each suggested the comparison of an amputation ; but only to further suggest that puerperal diseases were similar to those resulting from surgical wounds.

simply transferred from one position to another—and the analogy to the fruit of the tree is no longer observed. The former, instead of being suspended from the uterus, becomes suspended from the breast, and continues to be there nourished by the maternal blood. But that blood is now filtered through a gland cell, and the motion of the leucocytes is transmuted into the motion of milk corpuscles.

In the process of this amputation, and of the production of these two wounds, the survival of the excised part is analogous to the growth of plant cuttings and buds, after their implantation in a new soil, or their grafting upon a foreign stock. It is here that the pendulum of life swings most nearly out of the limits of physiology into those of pathology. Here most readily does a normal mode of motion become abnormal. The effect upon the mother of this sudden cessation of the vascular current setting in to the fœtal veins, is profound for good or evil. There is a temporary pause before the current again floods beyond its usual limits into the milk-stream.

Health, and what we call disease, touch each other never so nearly as during this interval. Ever so slight an interference with function at this time—ever so insignificant a derangement of force—may confer upon the trembling bioplasts a species of motion which may prove potentially destructive in an incredibly short space of time. It may actually beget a contagious disease, capable of extending itself throughout a community of human beings with fatal energy; may induce a series of changes which will permanently impair the economy of the organism; may even so add its effect to that of the ordinarily benignant maladies as to impart to them somewhat of its own destructiveness and contagiousness.

It is conceivable that the maternal leucocytes may be so influenced by the mutations described above, that they become capable of preserving, for a longer or shorter time, their novel modes of motion—of transmitting still further modified activities to their descendants—of extending numerous diseases to the other sex and to many individuals: in short, of becoming primordial "disease germs."

Do contagious diseases originate in this manner? Did they ever originate in such a manner? Are contagious diseases modified by such a process? These are interesting and important questions, which cannot perhaps, in the present state of our knowledge, receive a categorical answer.

A critical examination of the literature of puerperal disease will suggest to the candid and unprejudiced student, that, in the multiplicity of conflicting dogmas, much that is founded upon fact, much that is of itself incontrovertibly true, is embodied in each. And yet each seems to just fall short of the expression of a fact or principle, which might serve as a clue to the pathology of the disease.

Dr. Fordyce Barker, in his valuable treatise upon this subject, recently published in this country,* states that more than twenty-thousand pages have been printed in connection with puerperal fever during the last twenty years. Subjoined is a brief analysis of his careful review of the various theories respecting this disorder, which represents modern authors. According to Prof. Barker, "it occurs in a peculiar state of the system, arising from a modified condition of the blood, induced by gestation; from lesions of organs resulting from compression, contusion and laceration by the process of parturition; from a retrograde metamorphosis of uterine tissue; from the special physiological changes of the internal surface of the uterus; and from the function of lactation."

The first theory considered is that of the *localists*, who would have it appear that the fever and general symptoms are secondary to, and consequent upon, inflammation of one or more of the tissues and organs concerned in the parturient process. This is, or was, the opinion of Professors Meigs and Alonzo Clark, of our own country; Beau, Piorry, Jacquemier, Cazeaux, Trousseau, Behier, Mattei, Pijot, and Berne, of France; and Dr. Robert Lee, of Great Britain.

The second theory is that of Cruveilhier, Sir James Simpson, Spiegelberg, of Breslau; and Professor Schroeder, of Erlangen. These authors regard puerperal fever as analogous to traumatic fever, and the severer forms of it as due to either septicæmia or to pyæmia.

According to a third school of writers (Prof. Jos. M. Smith, Guerard, Dubois, Depaul, Danyau, Lorain, Tarnier, Fox, Ken-

* The Puerperal Diseases. Fordyce Barker, M.D. New York. 1874.

nedy, McClintock, and others), puerperal fever is primarily a blood disease, develoved, like other zymotic diseases, by epidemic, endemic and contagious causes, in which a modification of the general organism occurs, antecedent to the local lesions; consequently the local lesions are secondary: that is, they are the result of the disease, and not its cause. In short, according to them, it is an essential fever.

Dr. Tyler Smith, Dr. Robert Barnes, Professor Scanzoni, Braxton Hicks, Hall Davis, Graily Hewitt, W. S. Playfair, Wynn Williams, and Professor Leishman of Glasgow, hold to a fourth doctrine. They include under the term puerperal fever, all zymotic diseases, such as typhus and scarlet fevers, erysipelas, diphtheria, hospital gangrene, septicæmia, and all of the severe primary inflammations, when they occur in a puerperal woman. The idea of primary vitiation of the blood they do not reject, but term the disease a puerperal fever, whatever may be the nature of the specific primary poison.

Two authors, Professor Edward Martin, of Berlin, and Hervieux, of Paris, have adduced arguments in support of their several opinions, which differ from those given above. The former holds that "the diphtheritic process in the genitals of the lying-in-woman is the only essential element of puerperal fever." The latter believes that there is a plurality of puerperal diseases, as numerous as the local lesions, their type agreeing with the development of a puerperal poison—a sort of hospital miasm.

Prof. Barker's "confession of faith," after his investigation of the subject, is well worth reproduction. It is formulated as follows:

"1. There is a fever which is peculiar to puerperal women, and which is therefore appropriately named puerperal fever.

"2. The symptoms of this disease are essential, and are not the consequence of any local lesions, and it is as much a distinct disease as typhus, typhoid, or relapsing fever.

"3. It belongs to the class of zymotic diseases, and *results from some unknown blood changes.*

"4. *We are as ignorant of the specific cause of these changes* as we are of those which develop relapsing, scarlet, or any other of the essential fevers.

"5. The determining cause may be either epidemic influences, contagion, infection, or probably nosocomial malaria.

"6. Any of the local inflammations may occur in the puerperal woman without puerperal fever; and, on the other hand, puerperal fever may be so severe as to destroy life without sufficient local disease to account for the symptoms or explain the cause of death.

"7. The specific causes which develop the exanthemata, such as scarlatina or small-pox, may develop the specific disease with intense malignancy in the puerperal woman, but this does not transform the disease into a puerperal fever.

"8. Septicæmia may be developed in a puerperal woman either from autogenetic or heterogenetic infection, without puerperal fever, but this infection may also complicate puerperal fever."

The seventh and eighth of these propositions merely announce the non-identity of symptoms in puerperal and other disorders—a fact which is verified by daily experience. They by no means preclude the possibility of the existence of a causative or influential relation between zymotic diseases (with which puerperal fever is classed in the third proposition); and we are assured in the fourth proposition that the cause of these specific ailments is unknown.

Professor Leishman,* whose name has been already cited, says that "a series of facts of surpassing interest seem to show that the puerperal poison *may* be developed from other poisons of a similar kind, which has led some to conclude that the cause is less specific in its nature than a septic influence operating upon the peculiar conditions of the puerperal state." After illustrating this point by the citation of several cases, he proceeds to announce his belief, that the poison of the disease is associated with others of a similar nature. Attempts have indeed been made to prove that the poison of erysipelas is identical with that of puerperal fever, and although it is too much to assume that this has been made out, "*there can be no doubt that either affection is capable of being generated from the other.*"

The work of Dr. Minor, of Cincinnati, which has just issued from the press,† is exceedingly valuable as a means of esti-

* A System of Midwifery, inclnding Diseases of Pregnancy and the Puerperal State. Wm. Leishman, M.D. Philadelphia. 1873. Page 656.

† Erysipelas and Child-Bed Fever. Thomas C. Minor, M.D. Cincinnati, 1874. Page 119.

mating the relative extent to which puerperal fever and erysipelas prevailed in the United States during the census year of 1870. On the score of taste, many objections might be made to the literary standard of this brochure, and yet it would be singular indeed, if from the mass of statistics which the author has compiled, some valuable conclusions could not be deduced. That one in particular, which the compiler has reached, will no doubt receive the assent of those who have the patience to follow in his footsteps.

He thinks that "in any place where erysipelas is found, there will be found puerperal fever," because:

"1. Erysipelas and puerperal fever seem to prevail together throughout the States;

"2. Any marked increase in any one locality, of one disease, seems to be accompanied by a corresponding increase of the other;

"3. Where histories of past epidemics of either disease are obtainable from any of the States, the seeming connection of the two diseases was noticed by physicians at the time of such epidemics, and remarked on; and

"4. For these reasons we are, I think, justified in concluding that there is an intimate connection existing between puerperal fever and erysipelas."

It has been aptly said, that where an unusually long list of remedies has been recommended in the treatment of a single disease, it is presumable, either that the latter has an inherent tendency to limitation after a given interval, or that no one of the remedial agents is capable of exercising upon it a specific curative effect. And it may be equally suspected in the multiplicity of opinions respecting the etiology of a single disease, either that the exact truth may have been partially and imperfectly recognized by each observer, or else that it has been totally concealed from all. And in the instance of puerperal fever such a suspicion is confirmed by the only portion of the verdict of the profession at large which seems to have been unanimously rendered, viz.: "*unknown blood changes.*"

The most modern of the doctrines relative to contagion, is that enunciated by the distinguished histologist, Dr. Beale, of London; and, although it may be objected to his teachings, that they are as yet unsupported by sufficient evidence, yet, in the language of one of the ablest recent reviews of the germ theories of disease : "they are ingenious, plausible, and worthy of provisional acceptance till better are found."* Their application to the puerperal etiology is conspicuously satisfactory, though, so far as I am aware, Dr. Beale has not illustrated this point in his late essays.

According to the latter,† the active principle of all febrile contagious diseases is of animal origin, and to be found in the living matter of bioplasm. The bioplasts, or leucocytes, in this view, are the sole active agents in the phenomena of life: the repair of injury, waste, the repair of and the economical utilization of material. Once diverted in their active energy, they may become powerful for evil rather than potent for good. By degeneration they may retain the power of producing bioplasts like themselves; but these descendants are incapable of building up new and normal tissues, and merely form cell colonies which are capable of producing disease and the lesions of disease, and of retaining the power for an ill-defined period, even when situated external to the living animal.

Beale exhibits in his work, plates which represent the disease germs discovered by him in vaccine and variolous lymph, and in the secretions removed from animals infected with the cattle-plague. Many of these are viewed under an objective of one-fiftieth of an inch focal distance; and when these are contrasted with others representing the normal growth of cartilage and vascular tissue, inspected through the same media, the result is a most vivid realization of the activity of the blood-bioplasts in health and disease.

Dr. Hurd, though dissenting from the conclusions to which Beale has arrived, is yet willing to concede that they "accord with the doctrines of the modern German school of biology. All recognize the white corpuscles as the chief blood bioplasts possessing a wonderful constructive power, in health, and being the active agents in disease." The discoveries of Woodward regarding the migration of these leucocytes, have served but to stimulate and extend the development of these facts.

* On the Germ Theory of Disease. E. P. Hurd. Boston Medical and Surgical Journal, July 30, 1874.

† Disease Germs, Their Nature and Origin. Lionel S. Beale. London. 1872.

It is no objection to this belief, as urged by Dr. Hurd, that "all kinds of germinal matter look alike, and are undistinguishable by any characters which the microscope or chemistry can discover;" but rather one of the chief arguments by which to support its validity. Do not all germs resemble each other to a degree that has awakened the keenest activity in the minds of the advanced thinkers of our age? The germs which enfold the future organisms, whether of plants or animals, exhibit no feature which can serve as an indication of the future form, sex or even species of the individual. Huxley, much contradicted and more abused, is nevertheless an oracle of wisdom when he asserts that "one mass of non-nucleated protoplasm is undistinguishable from another. Why call one, plant; and the other, animal? The only reply is, that so far as form is concerned, plants and animals are not separable, and that in many cases it is a mere matter of convention whether we call a given organism an animal or a plant."*

What we choose to call health and disease do not differ from each other by as much as as do the kingdoms of animal and vegetable life. There is a unity of type and design, and a similarity of germ and organ, in animals and in plants; and the homology of fever is to be sought in the flush of the ruddy cheek of youth. Nay, more; since the stork is said to endure with impunity the venom of serpents, and the quail to thrive on a diet of hellebore,† it may be that, for the complete understanding of a strictly physiological process, it is requisite to solve some of the problems of pathology.

Dr. Beale figures in a manner most lucid to the eye the movement —amœbiform and slow—of the living bioplasts; but upon this essential evidence of their vitality, he does not lay as much weight as upon the proofs of their marvellous reproductiveness. Still, it is possible that upon the former, the entire argument respecting the *contagium vivum* will eventually be found to turn. Heat, light and electricity are now demonstrated to be merely modes of motion, interchangeable and intercurrent. At present the motion of the bioplast appears to be not only the condition of its vitality, but a measure of its reproductiveness. A change once effected

* Fortnightly Review, February, 1869.

† Sir Thomas Browne. Vol. 3, p. 358.

in the mode of that motion, must of necessity exert an influence upon the direction in which that energy is exerted. The forces which produce the foot-pound and the units of heat and light, are not more separate than those which result in the bioplasm of cartilage and the neoplasm of cancer. It is no more difficult to believe that a blood corpuscle can store up the momentum which will eventuate in small-pox, than to assent to the fact that the swing of a sunbeam centuries ago, is released from a fragment of carbon in the impulse which propels the piston of an engine to-day.

The trend of the strata exposed by modern research is altogether in one direction—unity of design, uniformity of law and economy of force and material. Man is born of the same energy which produces the bud of the torula, and dies a ter the manner of diseased wheat. We say that visceral ectrophy in a human being is anomalous, but plants exhibit this phenomenon when they have arrived at the maturity of normal development. The tulip exhibits the phases of a perfect life when its digestive organs are outspread to the sun and air; and it is not ashamed to expose to the eye of the world all its members and processes of generation. Even the so-called "monstrosities" are beautiful in their display of order in perversion. That which is most normal "is often an expression of the widest diversity from type; and even the brain has been called a parasite of the complex animal organism."*

The correlation of the vital and physical forces is no longer a question, but a demonstrated fact of science. Barker, Le Conte, Meyer,† and others, have ably shown that nerve force, muscle force and thought force are transmuted potential energies, measurable by the amount of heat stored up in the food which is digested. But here they have stopped. They have contented themselves with studying the complex velocities of whole muscles, whole brains and whole bundles of nerves, in the healthy animal. It is the province of medicine to study the modified velocities

* Nouv. Dict. de Med. et de Chir. Article, "Contagion."

† Barker. Correlation of Vital and Physical Forces. New Haven. 1871. Le Conte. Correlation of Vital and Physical Forces. American Journal o Science, Vol. II, No. xxviii, p. 305. November, 1859. Meyer. On Organic Motion and Nutrition. Extract from Essay in "Tyndall on Heat." New York. 1867.

which generate disease not only in gross textures, but in the bioplast and its brood of pestilential germs.

If, then, the leucocytes of the blood are so altered in the abnormal puerperal state that they actually beget a contagious disease—and if it is true, as many have asserted, that the latter is intimately associated with other, if not all other, contagious diseases, then perhaps the creed of some of the fathers of medicine may not be, after all, so very far astray. For example, Alphonse Le Roi believed that most of the causes which produce degeneration in the human family originate with the female. "With a view more effectually to follow the labyrinth of the human economy," he wrote, "it appears necessary to study that of the female: in this way, we may perceive more frequently and more certainly than in that of the male, the course and progress of disease. Moreover, the degeneration of the species always begins in nature with the woman." And even Boerhaave was not far from the truth when he wrote in 1690: "Several diseases owe their Birth to no other Cause than Pregnancy, whereof some do proceed from the menstruous Blood being hindered to separate because of the Closeness of the Womb, and the Fœtus not being yet able to consume the Quantity conveyed thither for its Nourishment."*

Because such a statement might be paraphrased, in the language of modern science, as follows:

"Several diseases originate exclusively from the puerperal state, and some, from the abnormal energy of the bioplasts of that blood which, in the non-pregnant condition, would have become menstruous. But the retention of the latter during pregnancy, enables them to communicate their velocities to the bioplasts of the fœtus, and an excess or diversion of these velocities, beyond what is requisite for the production of physiological effects, generates disease."

A curious and interesting illustration of the influence exerted by the puerperal condition upon contagious disease, is to be found in the history of the attempts to secure immunity from variola by the process of inoculation and vaccination.

The connection between variola and vaccinia is not yet clearly

* Translation of the Aphorisms of Boerhaave. The title-page of this venerable work is wanting.

established. Whether the sheep, the horse, the cow, or man, first exhibited the symptoms of the parent disease, and which race was first to contract it from the earliest infected individual, is a problem the difficulty of whose solution is self-apparent. Yet it must be admitted that human variola, transmitted through the system of the cow, undergoes a remarkable transformation.

Now it is not a little singular that most of the observers and more of the experimenters in this interesting field, have been obliged to confine themselves not only to the female of the bovine race, but even to the lactiferous female. Ceely, in the *Transactions of the Provincial Medical and Surgical Journal* (vol. 8, p. 299) speaks on this subject as follows:

"It is considered that the disease (vaccinia) is peculiar to the milch cow—that it occurs primarily while the animal is in that condition—and that it is casually propagated to others by the hands of milkers. But, considering the general mildness of the disease, the fact of its being at times entirely overlooked, and that its topical severity depends almost wholly on the rude tractions of the milkers, it would perhaps be going too far to assert its invariable and exclusive origin under the circumstances just mentioned: yet I have frequently witnessed the fact that sturks, dry heifers, dry cows and milch cows milked by other hands, grazing in the same pastures, feeding in the same sheds and in continuous stalls, *remain exempt* from the disease."

Seaton, Medical Inspector to the Privy Council, says, in regard to the same subject, "it particularly attacks milch cows; indeed, as a spontaneous disease it occurs *almost, if not quite, exclusively*, when the animal is in that condition. Young cows and milch heifers appear to be more subject to it than older cows." *

In the numerous attempts to produce vaccinia by variolous inoculation of these animals, the same general influence of the puerperal state is clearly evident. Dr. Seaton reports that all attempts to infect cows directly by repetition of Sunderland's experiments—as in England, by Mr. Ceely;† in India, by Mr. Macpherson, and by Mr. Lamb;‡ and on the Continent, at the veterinary schools in Alfort, Berlin, Weimar, Bergen, Dresden,

* Hand-Book of Vaccination. Ed. C. Seaton, M.D. London. 1868.

† Op. cit.

‡ Transactions Med. and Phys. Society of Calcutta. Vols. 6 and 8.

Kasan, Utrecht and Stockholm, were unsuccessful. For although both at Utrecht and Stockholm, a pustular or vesicular eruption appeared on those parts of the animals experimented upon, which were in immediate contact with the infected coverings, this was evidently of a local non-specific character.

But in 1836, Dr. Thiele, of Kasan, after several fruitless attempts to infect cows by inoculation of the variolous virus, succeeded finally, on several occasions, and produced a virus which was subsequently employed in the vaccination of more than three millions of individuals, and which survived seventy-five transmissions. This success he attributed to the precautions taken in the selection of animals for his experiments; and his previous failures he attributed to neglect of these precautions. I quote his own words (the italics are mine): "Cows should be selected from four to six years old, *which have recently calved;* the inoculation should be performed at the base of the udder."

It is a matter of wonder that so many able experimenters have neglected to observe the coincidence of spontaneous vaccinia and the puerperal state, and to avail themselves of a knowledge of this fact in producing variolation. After a somewhat careful examination of authorities, I am unable to refer to any records of spontaneous vaccinia in bulls; and but a few in which oxen were affected with the disease.

"Grease"—which has in numerous instances produced vaccinia in cows, and even typical vesicles in the human being which subsequently secured immunity from variola—seems to occur indifferently in geldings and mares. When the latter are infected, not only the pelvic extremities exhibit cutaneous lesions, but also the vulva and teats. It is, however, quite probable that sufficient observations have not been made to establish, definitely, the history of this disease, since it is apparent in the descriptions recorded, that grease has been confounded with other non-contagious disorders. Even so good an authority as Forester, declares that "the disease is caused by neglect, the horse being allowed to stand in the stable with cold and wet heels,"* a condition which, though favorable to the production of inflammatory disorders, could hardly, of itself, be capable of begetting a contagious disease.

Gamgee, in his exhaustive work on the Cattle Plague, alludes to

* On the Horse. Vol. II, p. 492. New York. 1857.

the influence of parturition upon the extension of that disease. He states that pregnant animals are readily infected, but not so readily as at the period of calving. In these cases there is more or less redness of the visible mucous membranes, which is especially noticeable in the vagina of cows; and the characteristic eruption invades the mammæ, the inside of the thighs, the back and the loins.

A discussion of the interdependence of the puerperal and all contagious diseases, would exceed the scope of this article. But that such a relation exists—intimate, if not essential and causative—is tolerably clear from the evidence. The eruptive fevers, phthisis (which is still considered contagious by Villemin and Guerin) and syphilis, are admitted to be grave complications of pregnancy, but unlikely to occur—syphilis excepted—until after its completion. The protection which is hence assumed to be conferred by the gravidity of the uterus, has been attributed to extraordinary activity on the part of the vital forces, which are presumed to be too intently engaged upon the building up of a new being, to exert themselves elsewhere, or in other directions.

But it is probable that, if such immunity exists, it depends upon some other causes; for the non-contagious diseases do not find in the pregnant female any obstacle to their inception and continued progress. Non-specific eruptions, which are unaccompanied by fever, pursue an uninterrupted career during the continuance of pregnancy, and many such are not amenable to treatment till delivery has occurred, when they tend to a spontaneous disappearance. This is precisely the reverse of what occurs in the eruptive fevers, which are more easily contracted after delivery, than before. Cazeaux, on the authority of Serres, reports that this is true of scarlatina.*

If the conclusions here suggested are sustained and accepted, it will not be extravagant to regard the condition of the woman who is pregnant as analogous to that of her husband who has the small-pox. By contact (or contagion) she has been placed in a physiological state, through the medium of a germ, a ferment, or a virus—call it what we may. In an etymological sense, it were wiser to call it a virus, for the vis is that which constitutes the

* Traite theorique et pratique de l'art des Accouchements. P. Cazeaux. Paris. 1867. Page 438.

essential element of all venom and virility. His disease was contracted in a similar way.

She forthwith proceeds to elaborate a new organism by the process of cell multiplication and segmentation; just as he who is infected, develops his poison. But that which she elaborates is not the ciliated cell which she received from her husband. It is that which she herself secreted from her ovary—fructified, it is true, by the former, but yet a scion of her own blood stock.* That which he multiplies, is the leucocyte of his own blood, fructified, changed in function, velocity, or reproductiveness, by the cell with which he was infected.

She develops her cells with all the activity of maternity, in the great emunctory of the uterus—he, his, in the smaller emunctories of the muco-cutaneous system.

His blood is profoundly affected, and there results a febrile movement: so is hers, and the mammary, renal and other glands exhibit their sympathies.

When the fruit of her womb is mature, she brings it violently into the world: in like manner his pustule bursts and discharges its contents. In the one case, and in the other, the microscope can discover nothing in these products but masses of growing cells.

The perfect product of her womb is a thing of life: the perfected product of his pustule is living matter.

Each process leaves in the human body an indelible record of its history.

The greatest fatality in each, occurs after the expulsion of the organized mass.

But after this expulsion, these cell masses are not wholly abandoned to external influences. The child, for a further space of time, is fed upon its mother's milk; the scab, for a short period, is fed by its imperfect connection with the subjacent tissues.

The contents of the pustule can, after incubation, excite in an unprotected individual, the entire chain of phenomena which make up the disease. The product of the womb can also, after its arrival at puberty, impregnate, or be impregnated by, another, and thus perpetuate itself.

* Martin Barry has seen the spermatozoid actually find its way into the ovum, through a minute perture, in the Aascaris Mystax. See Bennett's Text Book of Physiology. Philadelphia. 1873. Page 48.

These comparisons may appear fanciful to some, but if they have not a substratum of fact, the revelations of the last decade are as untrustworthy as the utterances of the Delphic oracle.

There are many other interesting questions which the consideration of this subject suggests. Such are the relations between puerperal disease and the septicæmic affections of the new-born child — the influence of certain morbid mental and physical states of the mother upon the milk which she secretes, and, in especial, the study of the toxicology of milk. This secretion is so closely connected with the puerperal state, that the interest which attaches to it, is largely derived from that fact. The bacteria which the microscope has detected in this fluid,* have never sufficed to sustain the theories which would account for its morbific agency. It is more than probable that whatever power for good or evil it possesses, it owes rather to the leucocytes of the blood which are its legitimate ancestors, than to any microscopic germs of foreign extraction.

A great French cuisinier† has inscribed upon one of the first pages of his treatise on his art, the following words: "A cook should be above prejudices." What a lesson is there for the scientist! We have an authentic record of articulate speech from only one dumb animal, and that was not when he found himself confronted by an angel of truth. It was only when a soi-disant prophet, blinded by his prejudices, attempted to force the animal against the obstacle in his path, and bruised himself in the effort.‡

Perhaps we shall, in our day, read an explanatory answer to the question propounded to himself by a thoughtful physician, in the retirement of his study upon the Via Sacra of ancient Rome:

"*An quis varioiis et peste simul laboret?*"

* Transactions of the Royal Microscopical Society of Great Britain. Vols. 3 and 5.

† Pierre Blot—who recently died.

‡ Numbers xxii, 28.

